# Gold nanoparticles synthesis and immobilization by atmospheric pressure DBD plasma torch method†

**DOI:** 10.1039/d3na00007a

**Published:** 2023-04-13

**Authors:** Andjelika Bjelajac, Adrian-Marie Phillipe, Jérôme Guillot, Yves Fleming, Jean-Baptiste Chemin, Patrick Choquet, Simon Bulou

**Affiliations:** a Luxembourg Institute of Science and Technology, MRT 28, Avenue des Hauts-Fourneaux L-4365 Esch-sur-Alzette Luxembourg

## Abstract

Herein, we report the impact of plasma on gold nanoparticles synthesis. We used an atmospheric plasma torch fed with an aerosolized tetrachloroauric(iii) acid trihydrate (HAuCl_4_·3H_2_O) solution. The investigation showed that using pure ethanol as a solvent for the gold precursor enabled a better dispersion compared to a water-containing solution. We demonstrated here that the deposition parameters are easy to control, presenting the influence of solvent concentration and deposition time. The advantage of our method is that no capping agent was used. We assume that plasma creates a carbon-based matrix around the gold nanoparticles preventing them to agglomerate. The XPS results revealed the impact of using plasma. Metallic gold was detected in the plasma-treated sample, whereas the no-plasma sample revealed only Au(i) and Au(iii) contributions originating from the HAuCl_4_ precursor. Detailed HRTEM, EDS mapping, and SAED analyses led to more insights into the structure.

## Abstract 2

Well dispersed Au NPs of narrow size distribution were obtained using an atmospheric DBD plasma torch. Ethanol used as solvent for Au precursor served as well as a C source for encapsulation of Au NPs preventing them from agglomeration.

## Graphical abstract

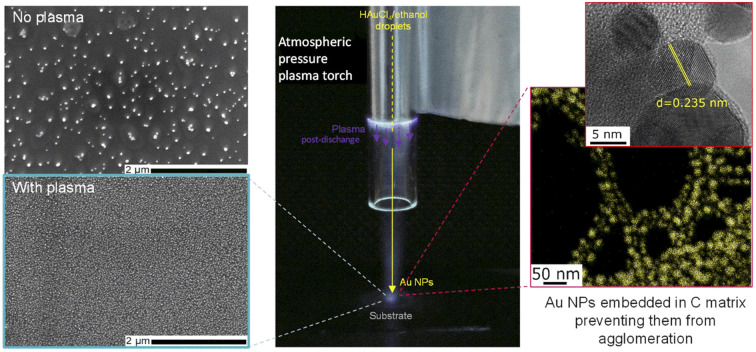

## Introduction

It was in 1857 when Faraday managed to synthetize gold (Au) nanoparticles (NPs) dispersed in water for the first time.^1^ This discovery set a highly prospective path of science and engineering of gold NPs. As NPs of gold are chemically inert towards (photo)oxidation, they found numerous applications, including medical therapy,^2^ drug delivery,^3^ chemical sensing,^4,5^ catalysis,^6,7^ and electronic applications due to the size and shape dependent surface plasmon resonance (SPR).^8,9^

The physical methods for gold NPs synthesis, *i.e.* the e-beam evaporation^10,11^ or laser ablation of a Au target^12^ provide high purity gold NPs. Yet, the preference is given to the chemical synthesis since it provides more NPs surface modification and consequently opens more application possibilities. These chemical routes originated from the Turkevich approach. The Turkevich method involves the use of sodium citrate for the reduction of chloroauric acid (HAuCl_4_) at elevated temperatures.^13^ However, with this method, it is difficult to control and predict the NPs size and morphology. Also, one of the disadvantages of the Turkevich method is the use of water as a solvent, because sodium citrate is practically insoluble in alcohol. In addition, the use of water as solvent limits the labeling of gold NPs by organic drug molecules, which are usually less soluble in water.^14^ Thus, attention is given to overcome these limitations of the Turkevich method by optimizing the reaction medium (type of Au precursor, ligands, *etc.*) and by controlling the properties of the solvent (*i.e.* using a mixture of water and ethanol^14^). Many studies proposed different methods, such as Brust–Schiffrin, where an aqueous solution of HAuCl_4_ was mixed with a toluene solution of tetraoctylammonium bromide (TOAB).^15^ Furthermore, some green methods^16,17^ using plant extracts are documented, but the procedure for plant extract preparation includes some extra preliminary steps. A more simple way to synthetize gold NPs is to use a stabilizer agent, commonly a bio-ligand, such as polyvinylpyrrolidone (PVP)^14^ or polydopamine (PDA)^18^ or poly(ethylene glycol) (PEG).^19^ In case of using a capping agent, the optimization of its concentration is required since it affects the NPs stability, size, and distribution.^20,21^ Additionally, Bouchard *et al.* pointed out the importance of 5–50 nm size range and spherical shape of Au NPs for medical application.^22^

One of the ligand-free methods for Au NPs synthesis was reported by Mariotti *et al.* where they applied plasma directly on the Au precursor solution.^23^ Herein, we investigate the creation of Au NPs by using an atmospheric pressure Dielectric Barrier Discharge (DBD) plasma torch fed with a nebulized Au precursor solution. It was previously demonstrated that DBD is one of several configurations of plasma torches that allows metallic NPs synthesis, as reported by Ghosh *et al.* where Ni NPs were synthetised by using nickelocene vapor.^24^ Our goal was to synthesize and immobilize the Au NPs of narrow size distribution that are well dispersed, without the use of ligand. Furthermore, Hussain *et al.* reported the effect of the solvent polarity on gold NPs synthesis by varying the ethanol-to-water ratio used as solvents for HAuCl_4_·3H_2_O. In their study, nearly spherical nanoparticles of 9.7 and 13.9 nm were produced by the Turkevich approach in 20 vol% and 50 vol% of ethanol-to-water solvent mixture, respectively.^14^ Thus, we also studied the effect of ethanol-to-water solvent ratio on the Au NPs synthesis.

## Experimental

### Synthesis of Au films

**Fig. 1:**
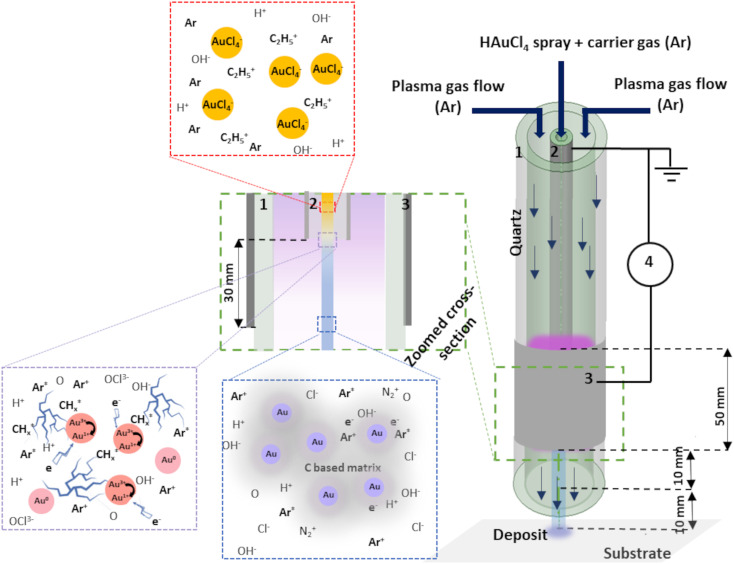
Schema of the experimental set-up: 1: outer quartz tube, 2: inner injection tube with Pt coating, 3: Al foil and 4: generator.

Tetrachloroauric(iii) acid trihydrate (HAuCl_4_·3H_2_O, >99.9%) bought from Sigma-Aldrich was used as the Au precursor by dissolving it in a solvent (ethanol and/or water). The injection of 100 μl min^−1^ was done using a Hamilton 10 ml syringe and a syringe pump system. The microdroplets were produced thanks to an ultrasonic nebulizer (Sono-Tek®, 1 W, *f* = 120 kHz). Laser diffraction analysis revealed that the droplets size ranged between 10 and 100 μm, whatever the composition of the solvent was. Ar was used as carrier gas (10 slm flow rate) to carry the aerosol into the plasma near post-discharge. The atmospheric plasma torch used here is based on a coaxial DBD geometry, composed of 2 concentrical hollow quartz tubes. The plasma is ignited between the inner tube (6 mm outer diameter, 4 mm inner diameter) and the outer plasma tube (9 mm outer diameter, 7 mm inner diameter) The inner tube (1) is coated on its outer surface with a 300 nm thick Pt film and connected to the ground. The Pt film was deposited using a Physical Vapor Deposition (PVD) technique. The outer quartz tube (2) was covered by a 5 cm long Al foil (3) connected to the HV generator (4). To produce the plasma, 10 slm of Ar is sent in the gap between the 2 tubes, and a sinusoidal HV is applied to the outer electrode (AFS generator, 52 kHz, 20 W). The inner hollow grounded tube is used to carry the nebulized Au precursor solution in the near post-discharge, 3 cm below the upper edge of the HV electrode. We chose this specific configuration to maximize the interaction between plasma post-discharge and the gold-containing solution droplets. The schema of the experimental setup is given in Fig. 1. To provide the insight of possible mechanism of Au NPs creation within the setup, an assumed scenario is also presented.

The substrates for the deposition were chosen according to the needs of the characterization technique, *i.e.*, Si wafer for scanning electron microscopy (SEM), X-ray photoelectron spectrometry (XPS) and X-ray diffraction (XRD), a 300 mesh Cu holey carbon grid for transmission electron microscopy (TEM), and a silica glass plate for optical measurements.

We investigated the influence of several synthesis parameters to study under which conditions the Au deposit would turn well dispersed and homogeneous in size:

(i) Concentration of the HAuCl_4_·3H_2_O was varied (0.025, 0.25, and 25 g l^−1^) but only in ethanol solution and for 10 min deposition.

(ii) Solvent composition: pure ethanol, ethanol: water mixture with 25 and 50 vol% of water, and pure water, 10 min, 0.25 g l^−1^.

(iii) Time of deposition: 1, 5, 10, 30, and 60 min only for precursor dissolved in ethanol, 0.25 g l^−1^.

### Characterization techniques

The SEM investigations were carried out on a Hitachi SU-70 FE-SEM, whereas TEM analyses were done on a JEOL JEM-F200 cold FEG microscope operating at an acceleration voltage of 200 kV. Crystalline nanostructures were analyzed by direct spacing measurements on High-Resolution TEM (HRTEM) images as well as by Selected Area Electron Diffraction (SAED) using Digital Micrograph Software from Gatan (version v.3.50.3584.0). Energy dispersive spectroscopy (EDS) mapping was done in STEM mode.

The transmission spectra were recorded using LAMBDA 1050 UV-vis-NIR spectrophotometer from PerkinElmer with a 100 mm integration sphere. Measurements were performed in the UV-vis spectral range (250–800 nm).

X-ray diffraction spectra were recorded at a fixed grazing incidence of 0.5° on a PAnalytical X'Pert Pro instrument equipped with a Cu Kα anode (1.54184 Å) and operated at 45 kV and 40 mA. A strain-size analysis (Rietveld method^25^ together with the Caglioti^26^ parameters previously determined using a NIST1976 Corundum Standard) was performed in Highscore Plus version 5.1b^27^ for determining the crystalline domain size of the gold phase deposit.

X-ray photoelectron spectra were acquired using a Kratos Axis Ultra-DLD photoelectron spectrometer with a monochromatic Al Kα source (10 mA, 15 kV) and a 700 × 300 μm spot size. Survey spectra were acquired using a pass energy of 160 eV, whereas high resolution spectra of the Au 4f, Cl 2p, C 1s, O 1s, and Si 2p regions were collected with a pass energy of 20 eV. The binding energies were referenced to the adventitious carbon at 285.0 eV. The shape of a reference spectrum acquired on a sputter-clean gold foil was used to fit the metallic Au 4f component. All other components were reconstructed using Gaussian–Lorentzian peaks after removing a Shirley type background.

## Results and discussion

### Varying the precursor concentration

**Fig. 2:**
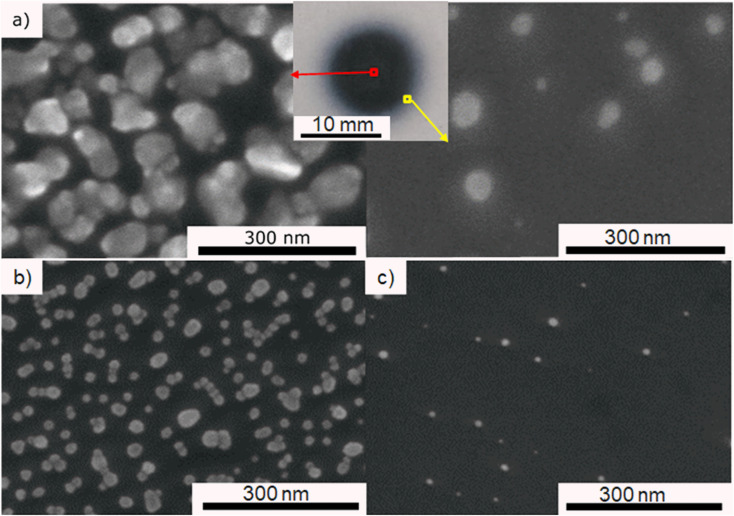
SEM micrograph of Au deposit after 10 min of plasma deposition using: (a) 25 g l^−1^ precursor (centre and the edge of the deposit), (b) 0.25 g l^−1^ precursor, center and (c) 0.025 g l^−1^ precursor, center.

In order to find the optimal precursor concentration to achieve narrow size distribution and good dispersion of the NPs, the first experiment was done using 25 g l^−1^ precursor solution in pure ethanol. A dense circular dark coating of approximately 10 mm diameter is obtained after 10 min of deposition with plasma applied under the fixed conditions given in Experimental section. SEM top view observations revealed that ∼100 nm agglomerates were obtained, and the representative micrograph is given in Fig. 2a. The corresponding histogram of size distribution in provided in ESI in Fig. S1a,† however the precise measurement of the individual particles was hard since most of them were agglomerated. The density of the deposit decreased from the center towards the edge (4 mm from the center), revealing smaller NPs of ∼10 nm. For the sake of comparison, all observations and particles characterizations were thus performed at the center of the samples. Since our objective was to obtain well dispersed NPs displaying narrow size distribution, 2 solutions of lower concentration (0.25 and 0.025 g l^−1^) of HAuCl_4_·3H_2_O in pure ethanol were prepared. With the same deposition parameters, less dense deposits were obtained. Fig. 2b and c are SEM micrographs taken at the center of the deposit using 0.25 and 0.025 g l^−1^ solutions of precursor. The corresponding histograms of size distribution are provided in Fig. S1.† As one can see on Fig. 2c, the 0.025 g l^−1^ solution provided a too low density deposit. The 0.25 g l^−1^ was, thus, chosen as the optimal concentration. All further investigations were done using that concentration of the precursor.

**Fig. 3:**
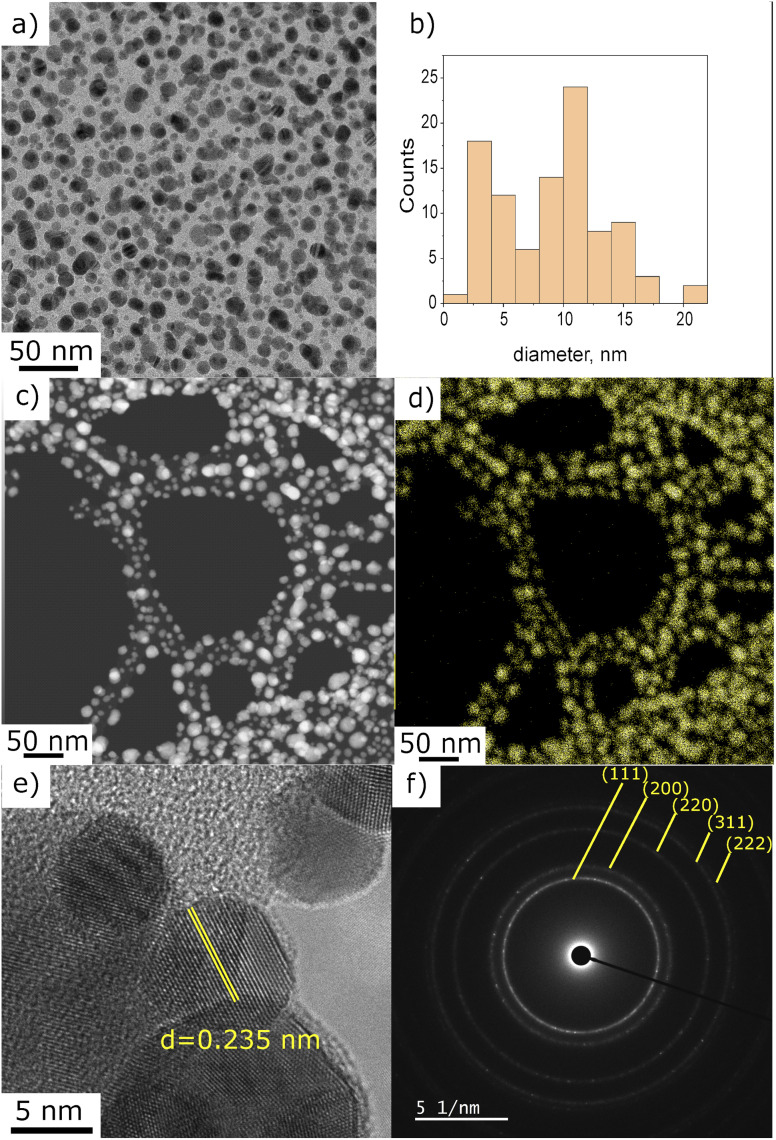
(a) TEM micrograph of Au deposit obtained using 0.25 g l^−1^ precursor in pure ethanol, 10 min of plasma deposition, (b) NPs size distribution histogram, (c) STEM micrograph and (d) corresponding EDS mapping, Au signal, (e) HRTEM with measured interplanar distance, and (f) SAED of the area given in (a).

For a more accurate determination of NPs size distribution, TEM analysis was performed on the deposit obtained using 0.25 g l^−1^ of gold precursor in ethanol solution after 10 min with applying plasma. This set of parameters enables the synthesis of NPs displaying a homogeneous dispersion, as illustrated in Fig. 3a. The size distribution histogram in Fig. 3b was obtained by measuring the size of 200 individual NPs. It is bimodal with a population of small particles displaying a size around 3 nm and larger ones close to 12 nm. The EDS mapping performed in STEM mode shows that the observed particles are made of gold (Fig. 3c and d). The chemical mappings of other detected elements (C, Cl, and O) are given in ESI Fig. S2.†

The interplanar distance of several NPs, measured on HRTEM images, matched well with metallic gold. Fig. 3e illustrates the interplanar spacing of 0.235 nm, which corresponds to the characteristic distance of (111) plane of metallic gold.^28^ In addition, the SAED analysis performed on the region presented in Fig. 1a is in good agreement with the Au pattern (JCPD-ICDD 00-004-0784 card). However, we also found (on one out of fifteen analysed NP) an interplanar spacing of 0.212 nm that can be associated to AuOCl (003) plane or AuCl_3_ (−241) or even AuCl (004). For one NP out of ten, we also measured a spacing of 0.56 nm, closely matching the (101) plane of AuOCl. Despite the unambiguous identification of metallic gold nanoparticles, several other gold-based materials were found to be present in the deposit. Even though we do not propose a full explanation of each of these species' formation mechanisms at this stage of the study, we assume that they are side-products of the plasma-induced synthesis or the result of oxidation paths occurring post-deposition. The possible mechanism will be the subject of a discussion further in the article.

### Influence of solvent composition

**Fig. 4:**
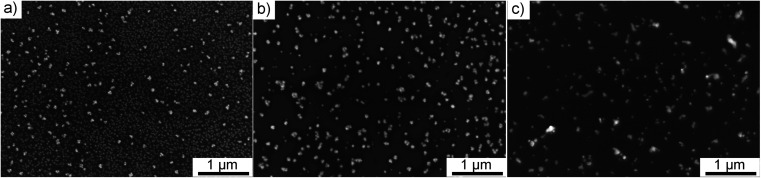
SEM micrographs of Au deposit obtained using 0.25 g l^−1^ precursor in: (a) ethanol–water mixture with 25 vol% of water and (b) 50 vol% of water, and (c) pure water for 60 min of plasma deposition.

Different ratios of ethanol : water were used with 0.25 g l^−1^ precursor concentration for 60 min plasma deposition to investigate the influence of solvent composition. By comparing the SEM micrographs (Fig. 4), it was noticed that the agglomeration tendency increased with the increase of water content. Similar as Hussain *et al.* explained,^14^ we show that using a higher percentage of ethanol in the solvent, meaning higher polarity, produces smaller and more spherical nanoparticles. In contrast, water-dominant solvents, meaning a lower polarity, result in larger gold NPs with different shapes. Therefore, we decided to focus on pure ethanol precursor solution for the rest of the study.

### Impact of the deposition time

**Fig. 5:**
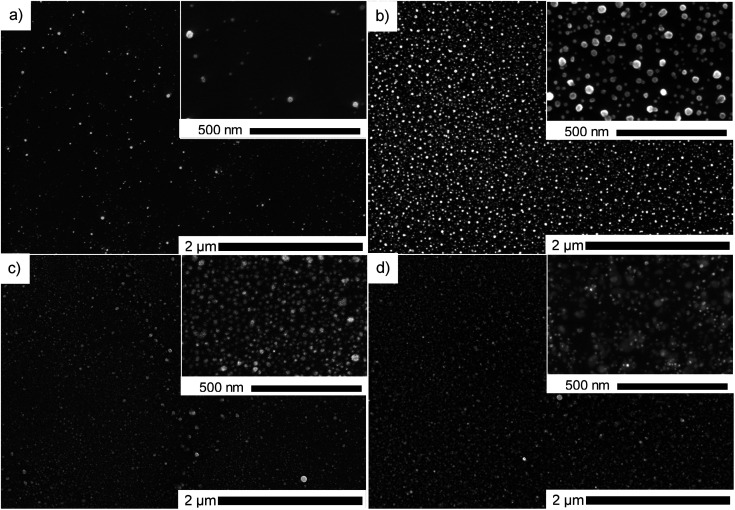
SEM micrographs of Au deposit obtained using 0.25 g l^−1^ precursor in pure ethanol, center, for various deposition duration: (a) 1 min, (b) 5 min, (c) 30 min and (d) 60 min.

Using gold precursor at 0.25 g l^−1^ in pure ethanol, we investigated the impact on NPs of various deposition time ranging from 1 to 60 min. All the presented micrographs were acquired on the specimen area localized at the center of the deposition. Fig. 5 shows that the density of NPs changed upon increasing deposition time. However, the primary size of NPs did not show any dependence on this parameter. It is striking to see that, in all presented cases, the dispersion state of NPs was fully preserved. We attribute this feature to the presence of a carbon-based matrix surrounding NPs that prevents them from agglomeration. It is important to note that this carbon matrix was only observed when pure ethanol was used as a solvent for Au precursor.

**Fig. 6:**
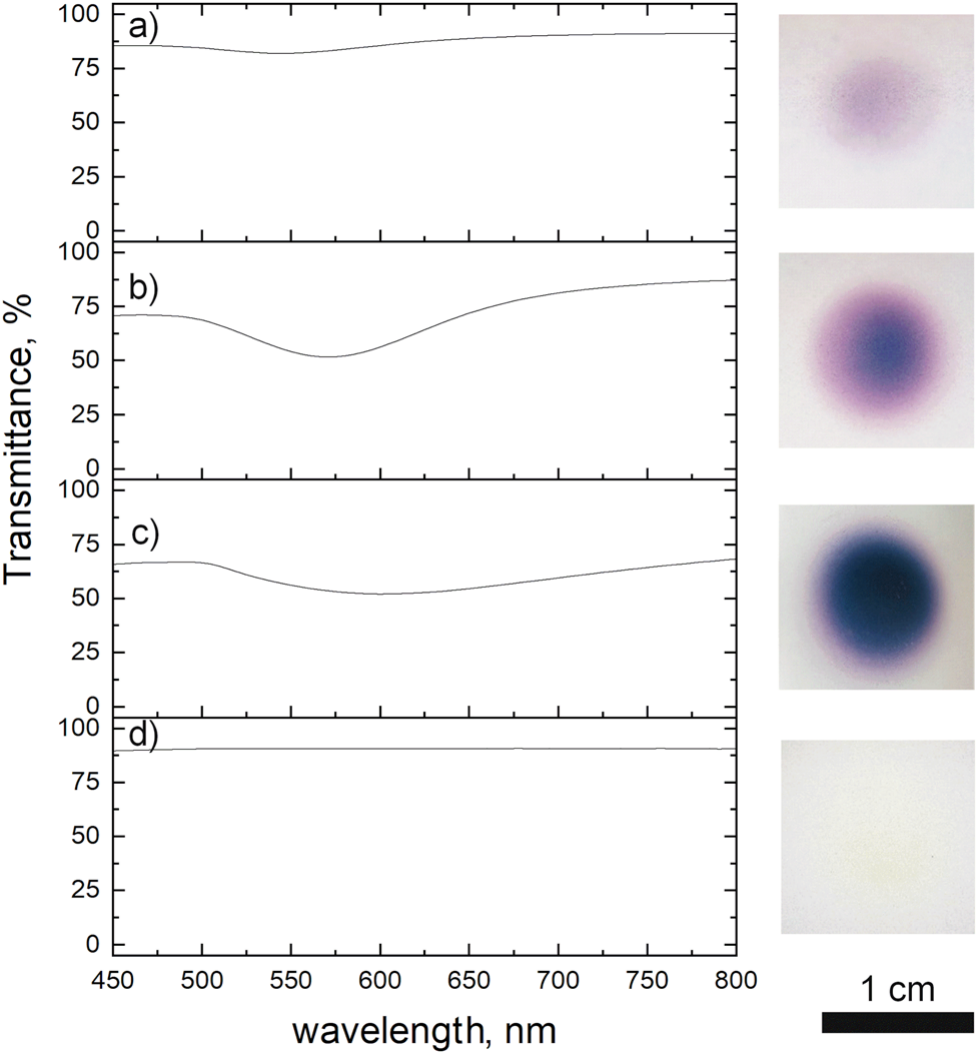
Transmittance spectra of Au deposit obtained after: (a) 10, (b) 30 and (c) 60 min of plasma deposition using 0.25 g l^−1^ ethanol precursor, (d) 60 min deposition without applied plasma.

We investigated the influence of deposition time on the optical properties of the deposit. The transmission spectra are presented in Fig. 6, together with the photographs of the samples. Due to their too low thickness, the 1 and 5 min deposits did not show any absorption peak. We thus, studied only the films obtained after 10, 30 and 60 min of plasma deposition. The 10 min film showed an absorption peak at 545 nm, which corresponds to the surface plasmon resonance of gold NPs.^23,29^ In the case of 30 min film, a red shift was noticed (571 nm). We explain this latter by the increase of the film density and consequently some NPs close to one another are merging and making one bigger (Fig. 5c). This merging of gold NPs was observed before by Sutter *et al.* under the *in situ* TEM^30^ where they stated that only NPs closer than 1 nm are subjected to that. Others remained separated. The same effect we notice for the 60 min deposit (Fig. 5d). This film having an intense bright yellow colour, showed an absorption peak at 601 nm. The plasma influence is unambiguously evidenced in the transmittance spectrum of the film obtained after 60 min of deposition. Using the same precursor and injection parameters but without plasma and for the same time, there was no absorption peak observed as the thickness of the film was very low. SEM micrograph of deposit obtained after 1 h without applied plasma is given in the ESI Fig. S3,† where one can see that, indeed, the deposit is of low thickness, and its distribution looks to be disordered.

### Plasma role in the Au NPs synthesis

**Fig. 7:**
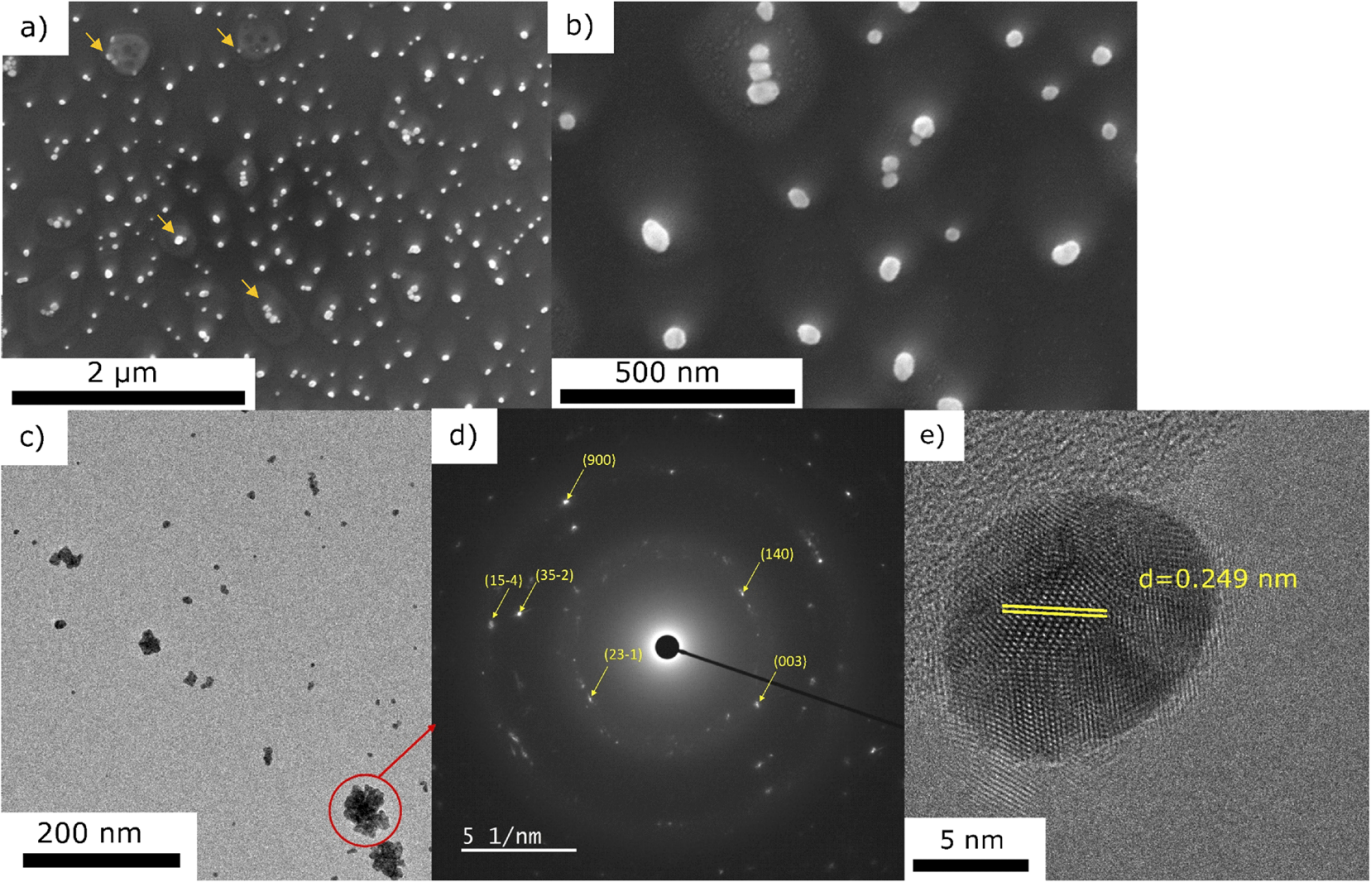
Representative SEM (a and b), and TEM micrographs (c) with the corresponding SEAD (d) and a HRTEM micrograph (e), of Au containing deposit obtained using 0.25 g l^−1^ precursor in pure ethanol without applying plasma, 10 min.

To get an insight into the mechanism of the Au NPs nucleation and growth, we performed the same deposition (0.25 g l^−1^ in pure ethanol for 10 min) without plasma. The corresponding SEM and TEM micrographs are presented in Fig. 7. One can observe the remaining of dried droplets from the SEM micrographs (Fig. 7a). There are NPs of ∼50 nm coming from the droplets drying, either in droplets' center or at the border (marked with arrows on Fig. 7a and b). The TEM micrographs revealed two kinds of morphologies: the agglomerates (like the one circled in Fig. 7c) and the individual NPs (such as the presented one given in Fig. 7e). Compared to the deposition obtained with plasma (Fig. 3), one can clearly see that the nanoparticle density is much lower without plasma. Moreover, NPs agglomerates are observed, whereas they were not seen for the deposition obtained with plasma. SAED analysis of one of these agglomerates is presented in Fig. 7d. By exploring the different JCPD-ICDD cards (obtained experimentally or calculated), a close match was found with gold oxide chloride (JCPD-ICDD 04-012-8341 card^31^). In addition, the HRTEM image of one NP (Fig. 7e) shows, for instance, the 0.249 nm interatomic distance corresponding to the (23−1) plane of gold oxide chloride. SEM-EDS analysis performed on the area displayed in Fig. 7b (data not given since there is a high signal of Si substrate with respect to other peaks) showed the significant presence of Cl together with Au, with the ratio Au/Cl = 5.04, whereas in the case of the plasma treated sample the amount of Cl was negligible. Therefore, we assume that the NPs are spontaneously formed just by precursor droplets' drying, as similarly discovered by Lee *et al.*^32^ However, they used water as a solvent and sodium borohydride as a reduction agent. Herein, we propose the following scenario of Au structures formation: the droplets are drying, and NPs are created in both cases, with or without plasma. Such spontaneous formation was explained by Lee *et al.*^32^ as a consequence of a strong electric field at the water–air interface of the microdroplets. In their study, there were also both agglomerates as well as individual NPs, like seen in our study in the case of no-plasma deposition (Fig. 7). It is only in the case of plasma that the NPs are well dispersed, which we believe it is due to a carbon matrix created on the surface of Au NPs originating from ethanol. It is also due to plasma that the amount of Cl reduces just by simple evaporation from the system. Without plasma, different oxides and chlorides can be formed,^32^ like AuOCl, which was detected in our case.

**Fig. 8:**
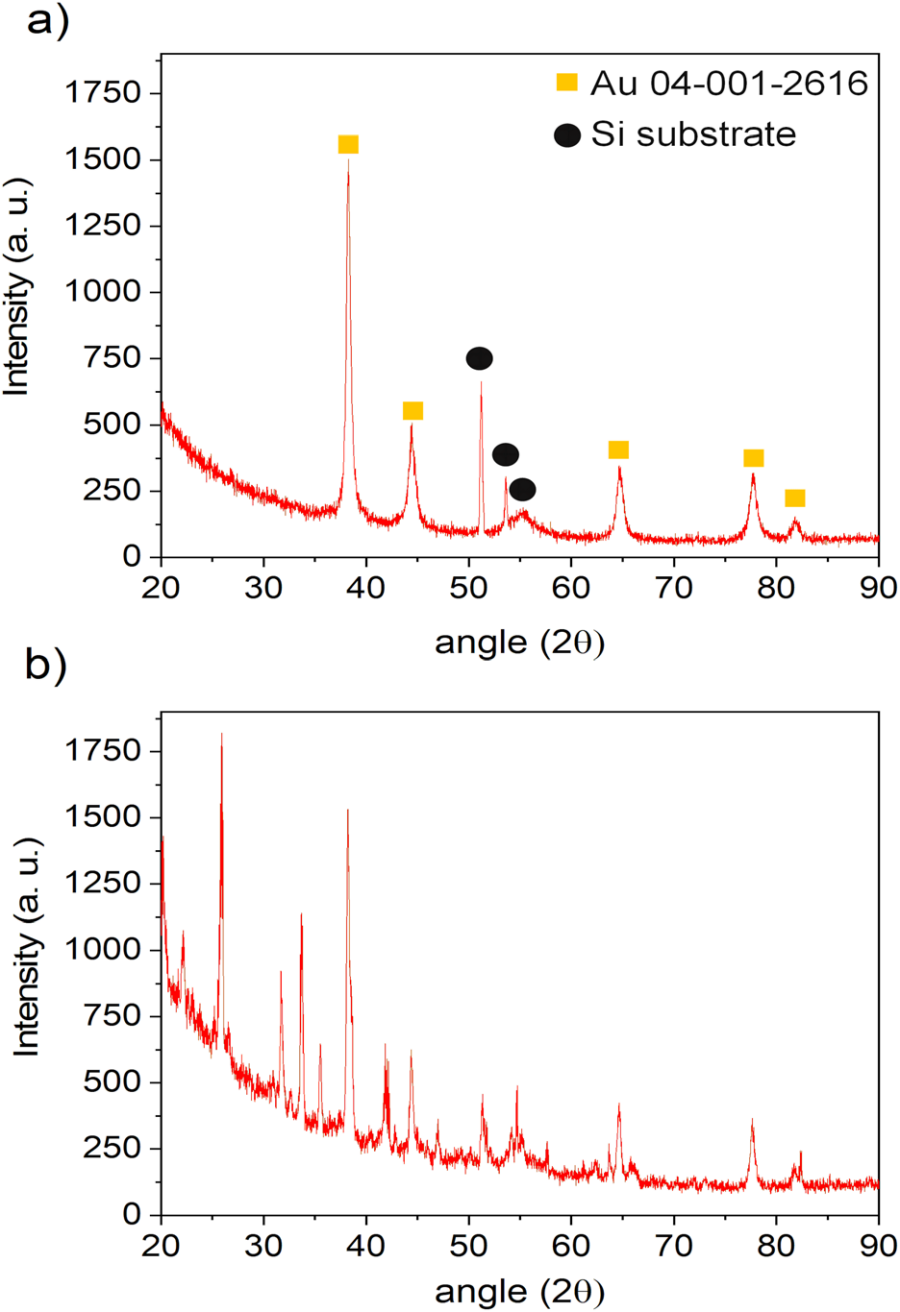
XRD patterns of the samples obtained by: (a) 1 h of deposition with applying plasma and (b) 10 h of deposition without applying plasma, both using the same precursor solution and injection conditions.

The XRD analyses were performed on both of the samples, with and without plasma treatment during the deposition step, using a 0.25 g l^−1^ precursor in ethanol solution. The depositions were done 1 h for plasma-deposited sample and 10 h for no-plasma deposition to ensure the good coverage of the substrate and to increase the signal of the deposit. The results are given in Fig. 8. One can see that in the case of no-plasma sample the composition of the deposit was very heterogeneous, whereas in the case of plasma deposited sample, only the peaks for pure Au were detected as well as of Si substrate. The crystalline size of Au deposit was determined to be 21.4 nm and the micro-strain 0.338 ± 0.009%.

**Fig. 9:**
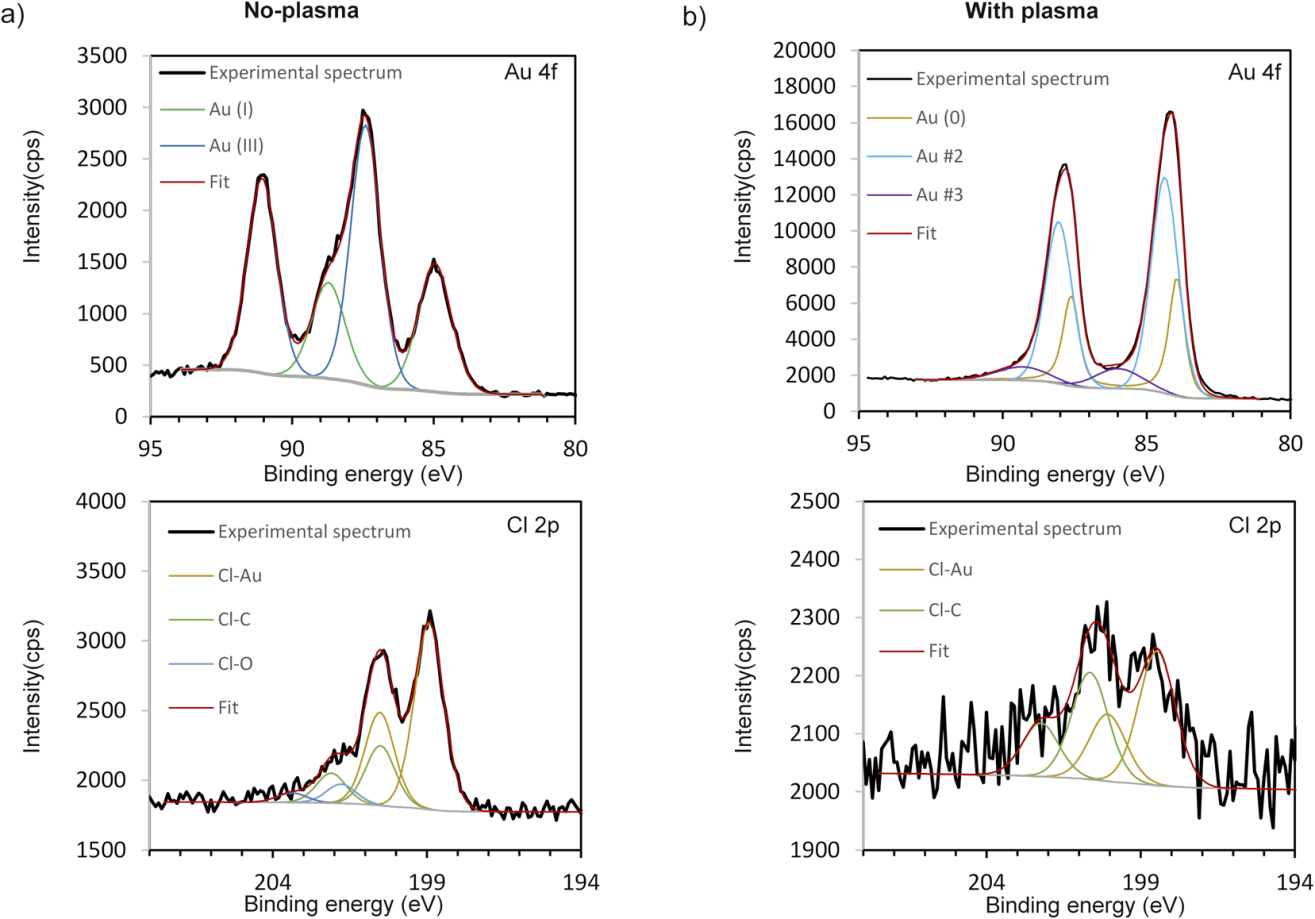
High resolution XPS spectra of Au 4f and Cl 2p envelopes of the deposits obtained: (a) without plasma and (b) with applying plasma during deposition, 10 min.

XPS analyses were carried out to determine the chemical states of Au and Cl to get more insight into the molecular structure of the obtained deposits (without and with plasma applied, Fig. 9a and b, respectively). High resolution XPS spectrum of the Au 4f 7/2 of the no-plasma sample shows 2 components corresponding to Au(i), at 84.9 eV, and Au(iii) at 87.4 eV.^12,13,33,34^ Recalling the results of TEM and SAED analyses, we can assume that Au(iii) component originates from AuOCl, whereas the Au(i) signal may be from AuCl. This was expected since it is well known that the precursor, HAuCl_4_·3H_2_O, is metastable and is prone to be reduced.^35^ However, in the case of plasma-treated sample, 3 contributions are identified as follows: the component at 84.0 eV matches well to bulk metallic Au and corresponds in this sample to Au(0) clusters electrically connected to the substrate.^36^ The contribution at 84.4 eV, so with a +0.4 eV upward shift, can be associated to metallic Au(0) nanoparticles of less than 2 nm as described elsewhere,^37,38^ but not detected on the TEM images. However, it can also be due to metallic Au(0) particles embedded in a carbon matrix and presenting a higher surface potential due to a low conductivity of the system and a too low electron refilling rate after the photo-ionization process.^39,40^ Gold–metal interaction with charge transfer was also previously described. Indeed, C-based capping agents could lead to the injection and trapping of electrons from Au NPs to the C matrix, which causes a downward shift of binding energy of C 1s as well as an upward shift of Au 4f and thus, decreasing the C 1s − Au 4f energy gap.^41–43^ The downward shift of C 1s cannot be evidenced as the peak was used for the energy calibration of the spectra. However, a decrease of the C 1s − Au 4f energy gap of 0.4 eV is observed. The first hypothesis (nanoclusters of less than 2 nm) does not seem realistic since comparing the intensity of the two main Au 4f_7/2_ components would mean that nanoclusters represent most of the gold amount deposited at the surface, which is not the case. The third and the smallest contribution at 86.2 eV can be associated to Au(iii), which is shifted downward. This tiny contribution may be coming from AuOCl NPs, as it was detected with HRTEM (Table 1). However, this contribution can also originate from Au_2_O_3_ or Au(OH)_3_ or AuCl_3_ which are in contact with metallic Au, as already reported in literature.^44–46^ Given the rather large contribution in the peak reconstruction (FWHM = 2.6 eV), we can also expect the coexistence of several phases. When a metal oxide is in contact with Au, a downward shift of the peak of the metal associated to that oxide is noticed, as already reported for TiO_2_,^47,48^ NiO^49^ or CeO_*x*_.^50^ To verify our hypothesis of Au 4f positions shift due to the C matrix around the NPs, we performed the XPS also on a deposit obtained using the same conditions but with the gold precursor dissolved in water. Indeed, the results showed that Au 4f position corresponds to Au(0) contribution only (ESI Fig. S4†), without any shift, as expected since without ethanol as a solvent, there was no C source for the creation of matrix around Au NPs.

**Table 1:** XPS elemental quantification from high resolution spectra

Sample	Au 4f, %	C 1s, %	Cl 2p, %	O 1s, %	Si 2p, %
No plasma	2.2	39.4	5.3	24.9	28.2
With plasma	8.8	49.6	1.0	31.8	8.8

The high resolution XPS spectra of the Cl 2p region of the no-plasma and plasma-created sample are also presented in Fig. 9a and b, respectively. For both samples, one can observe Cl 2p components at ∼198.5–199.0 and ∼200 eV, characteristic for metal and organic chlorides, respectively.^51^ The first contribution is attributed to Cl–Au (and/or Si–Cl, where Si is the substrate), whereas the second originates from C–Cl bonds.^52^ However, in the case of the no-plasma deposit, a small additional doublet is observed at 201.8 eV and can be associated to O–Cl bonds.^53^ This is in accordance with the TEM/SAED results where AuClO was identified in the case of deposition in the absence of plasma. The Cl/Au at% ratio (as deduced from XPS measurements) was found to be equal to 2.70 and 0.15 for no-plasma and plasma-treated sample, respectively (Table 1). This, again, tends to prove the major role played by the plasma in (i) enhancing the degradation of the precursor and (ii) promoting the formation of metallic nanoparticles.

According to our experimental findings, it is likely that, thanks to the introduction of precursor in the immediate vicinity of the discharge, an important part of the Au ions reduction may result from the interaction with plasma-produced free electrons. Besides, optical emission spectroscopy observation of the plasma discharge points out the production of highly reactive species and radicals such as OH, CH, C_2_ (ESI, Fig. S5†). Thus, we can assume that plasma-produced hydroxyl groups are involved in redox reaction in which Au(iii) and Au(i) were completely reduced to zero-valent Au(0). Similarly, it was suggested by Dursun *et al.*^54^ that the hydroxyl groups from covalent-organic polymer networks acted as a reductant for Au^3+^ ions.

We report here the strong plasma effect in providing a fine dispersion of Au NPs, embedded in a carbon-based matrix. On the contrary, the deposition without plasma is very scarce, and undesired agglomeration is present. Therefore, fabricating different composites that include Au NPs can be foreseen with the plasma deposition method described here. Our work provides a new pathway in Au NPs synthesis since the excellent dispersion of the deposit was enabled without any capping agent.

## Conclusions

Herein, we proposed a single-step synthesis of well dispersed gold NPs using a nebulizer and an atmospheric plasma torch. We showed how the different parameters, *i.e.* precursor (HAuCl_4_·3H_2_O) concentration, deposition time, and solvent composition influence the Au NPs dispersion and morphology. This study shows that the light response can be tuned and enhanced by engineering the shape and size of the Au NPs since the SPR property highly depends on such structural parameters.

Furthermore, we provided insight into the mechanism of the plasma created Au NPs. Using a pure ethanol-based solution with plasma during the deposition, a carbon-based matrix around the Au NPs was created. This C matrix has a significant role in preventing the Au NPs from agglomerating. The agglomeration tendency was higher when using a water-based solvent, not having the C source. XPS analyses confirmed that plasma induced the complete reduction of Au(iii) and Au(i) to Au(0) and a formation of C–Cl bonds.

## Author contributions

Andjelika Bjelajac: conceptualization, investigation, methodology; Adrian-Marie Phillipe: formal analysis, investigation; Jérôme Guillot: formal analysis, investigation; Yves Fleming: formal analysis, investigation; Jean-Baptiste Chemin: formal analysis; Patrick Choquet: funding acquisition, resources; Simon Bulou: funding acquisition, investigation, methodology, project administration, supervision, validation.

## Conflicts of interest

There are no conflicts to declare.

## Supplementary Material

NA-005-D3NA00007A-s001
