# Supplementary material for: Gold nanoparticles synthesis and immobilization by atmospheric pressure DBD plasma torch method
Source: Nanoscale Adv. 2023 Apr 13;5(9):2573–82. doi: 10.1039/d3na00007a (PMC10153074; doi:10.1039/d3na00007a)
Supplement: NA-005-D3NA00007A-s001 [file NA-005-D3NA00007A-s001.pdf]

Electronic Supplementary Material (ESI) for Nanoscale Advances.
This journal is © The Royal Society of Chemistry 2023

Gold nanoparticles synthesis by atmospheric plasma torch method

Andjelika Bjelajac[1], Adrian-Marie Phillipe[1], Jérôme Guillot[1], Yves Fleming[1], Jean-Baptiste Chemin[1], Patrick Choquet[1], Simon Bulou[1]

[1] Luxembourg Institute of Science and Technology, MRT, 28, avenue des Hauts-Fourneaux, L-4365 Esch-sur-Alzette

Supplementary file

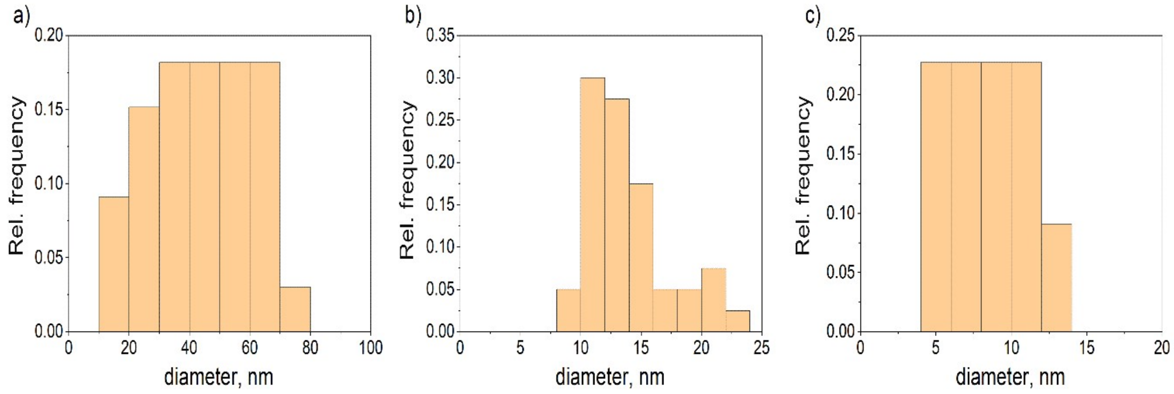

Fig. S1 Histograms of Au deposit (center) after 10 mins of plasma deposition using: a) 25 g/l, b) 0.25 g/l, and c) 0.025 g/l precursor,

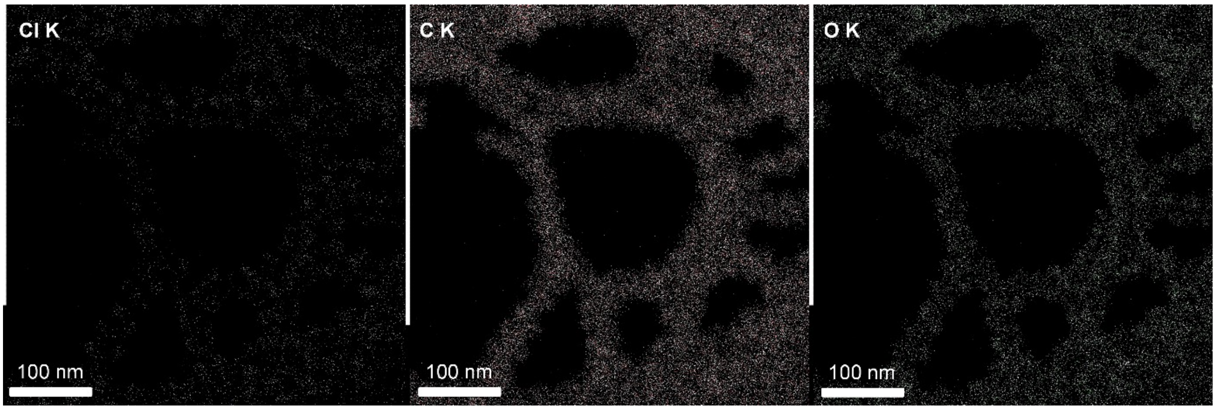

Fig. S2 Chemical elemental mappings of Cl, C and O obtained in STEM mode performed on a 10-min deposition with applying plasma sample

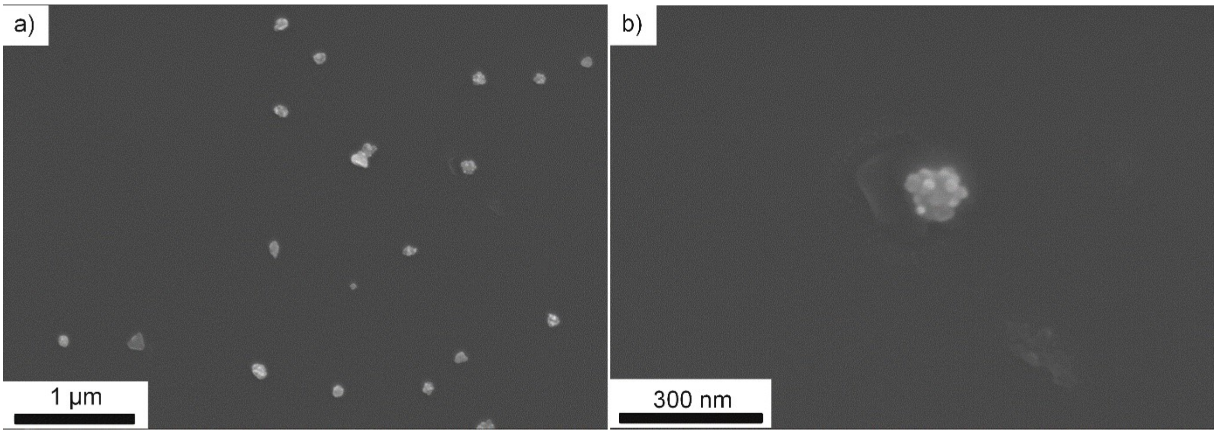

Fig. S3 SEM micrographs of a deposit obtained after 1 h of deposition without applying plasma

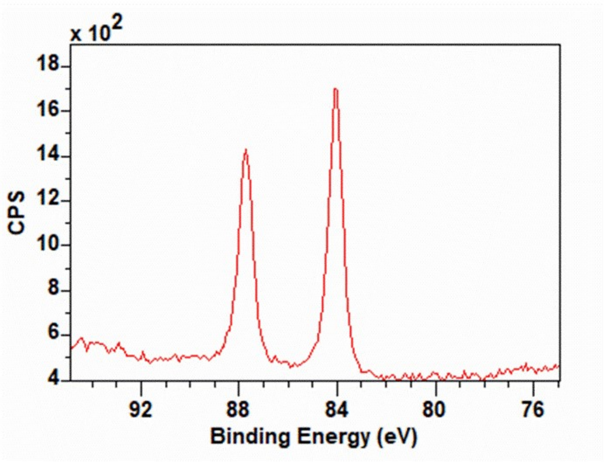

Fig. S4 High resolution XPS spectra of Au 4f envelope of the deposit obtained after 1 h of deposition using H_2O as a solvent for gold precursor, with applying plasma during deposition

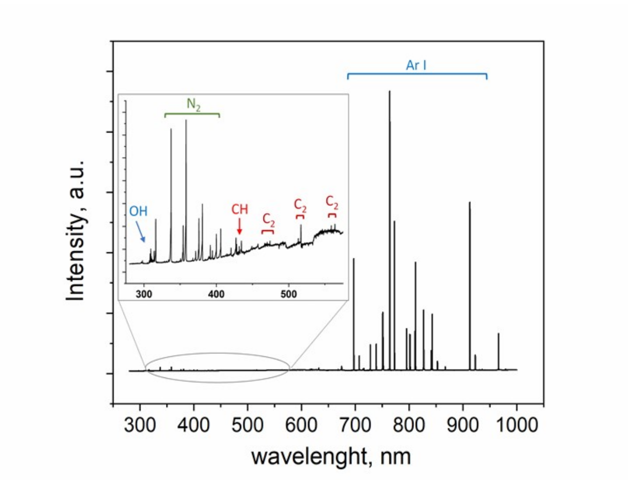

Fig. S5 OES spectrum of plasma discharge (Ar, 20 slm) with the droplets of 0.25 g/l Au precursor dissolved in ethanol
